# Leptin induces cell migration and invasion in a FAK-Src-dependent manner in breast cancer cells

**DOI:** 10.1530/EC-19-0442

**Published:** 2019-10-31

**Authors:** Juan Carlos Juárez-Cruz, Miriam Daniela Zuñiga-Eulogio, Monserrat Olea-Flores, Eduardo Castañeda-Saucedo, Miguel Ángel Mendoza-Catalán, Carlos Ortuño-Pineda, Ma Elena Moreno-Godínez, Sócrates Villegas-Comonfort, Teresita Padilla-Benavides, Napoleón Navarro-Tito

**Affiliations:** 1 Facultad de Ciencias Químico Biológicas, Universidad Autónoma de Guerrero, Guerrero, México; 2 Instituto de Fisiología Celular, Universidad Nacional Autónoma de Mexico, Mexico City, México; 3 Department of Biochemistry and Molecular Pharmacology, University of Massachusetts Medical School, Worcester, Massachusetts, USA

**Keywords:** leptin, FAK, Src, cell migration, metalloproteases, invasion, breast cancer

## Abstract

Breast cancer is the most common invasive neoplasia, and the second leading cause of the cancer deaths in women worldwide. Mammary tumorigenesis is severely linked to obesity, one potential connection is leptin. Leptin is a hormone secreted by adipocytes, which contributes to the progression of breast cancer. Cell migration, metalloproteases secretion, and invasion are cellular processes associated with various stages of metastasis. These processes are regulated by the kinases FAK and Src. In this study, we utilized the breast cancer cell lines MCF7 and MDA-MB-231 to determine the effect of leptin on FAK and Src kinases activation, cell migration, metalloprotease secretion, and invasion. We found that leptin activates FAK and Src and induces the localization of FAK to the focal adhesions. Interestingly, leptin promotes the activation of FAK through a Src- and STAT3-dependent canonical pathway. Specific inhibitors of FAK, Src and STAT3 showed that the effect exerted by leptin in cell migration in breast cancer cells is dependent on these proteins. Moreover, we established that leptin promotes the secretion of the extracellular matrix remodelers, MMP-2 and MMP-9 and invasion in a FAK and Src-dependent manner. Our findings strongly suggest that leptin promotes the development of a more aggressive invasive phenotype in mammary cancer cells.

## Introduction

Breast cancer is the most common invasive neoplasia and the second-leading cause of cancer-related death in women worldwide. Over 2,000,000 cases are diagnosed annually worldwide, which represents the highest incidence of cancer in the world (1). Also, obesity is a known risk factor for initiation, growth, invasion, and metastasis in breast cancer (2). Metastasis is a complication of cancer in which neoplastic cells escape from the primary tumor and develop secondary tumors in distant organs (3). This process involves loss of cell-cell junctions and cell-extracellular matrix (ECM) interactions, acquisition of migratory capacity, matrix metalloproteases (MMPs) secretion, degradation of ECM and release from the basement membrane which leads to cell invasion (3). Importantly, obese female patients present larger and more aggressive metastatic tumors to lymph nodes, than non-obese patients (4). Obesity is characterized by an increase in adipokine production, which further enhances the predisposition of developing breast tumors (4). Among these molecules, leptin is one of the most important adipokines involved in the development and progression of mammary tumors (5).

Leptin is a hormone with a molecular weight of ~16 kDa, encoded by the* LEP* gene located on human chromosome 7 (6). It is synthesized and secreted mainly by adipocytes, and in a smaller proportion, by the placenta, stomach, fibroblasts, skeletal muscle, and normal or tumorigenic epithelial mammary tissue (7). One of the primary functions of leptin is the regulation of food intake and energy expenditure, acting primarily through the hypothalamus (8). Leptin also regulates reproductive, immunological and metabolic functions (9). Additionally, leptin is involved in the progression of breast cancer, through the activation of mitogenic, anti-apoptotic and metastatic pathways (2). Leptin exerts these effects through the binding to the ObR receptor, activating various cellular signaling cascades such as JAK-STAT, MAPK and PI3K-Akt (7). Recent evidence showed that leptin levels in the plasma are higher in breast cancer patients compared with healthy individuals (2, 10). Furthermore, leptin and its ObR receptor are overexpressed in primary and metastatic mammary tumor tissues, suggesting an autocrine signaling mechanism developed by tumor cells (11).

Importantly, leptin seems to be related to breast cancer risk in premenopausal obese women, however, controversy exists (12). For instance, epidemiological analyses performed by the World Cancer Research Fund and the American Institute for Cancer Research from data up to 2017 showed that being overweight or obese decreased the risk of premenopausal breast cancer (12). Meta-analysis of pre-menopausal patients showed a reduced risk per 5 kg/m^2^ increase in the BMI (13). Thus, it was proposed that the pathophysiology between obesity and reduced breast cancer risk in pre-menopause women may be associated on their systemic high levels of estrogens, which in turn reduce gonadotrophin release, and decreased progesterone levels, thus reducing cell proliferation in mammary glands (14). Contradictory studies in this regard have proposed that progesterone may be protective against breast cancer (14). Studies in various populations have shown modest relationships between BMI, obesity and potential to develop breast cancer (15). On the other hand, studies in post-menopausal women showed that obese postmenopausal women presented increased risk for breast cancer compared to non-obese patients; furthermore and the degree of obesity has been correlated to larger tumors and metastasis (16). These patients are characterized by presenting with estrogen (ER-) and progesterone receptor (PR)-positive breast cancers, and not to ER-negative and triple-negative tumors (16). Thus, the effect of increased weight and BMI, as well as the role of leptin and the potential molecular mechanisms by which it contributes to breast cancer progression still remains to be elucidated.

The focal adhesion kinase (FAK) participates in the formation of focal adhesions and activates signaling pathways related to proliferation, survival, cell migration, and angiogenesis (17). Classically, FAK is activated during the formation of focal adhesions, and it is mediated by the interaction between ECM with β-integrins, triggering conformational changes in these receptors (18). This effect is followed by the autophosphorylation of FAK at Y397, which creates a high-affinity binding site for the Src-homology 2 (SH2) domain of Src, a non-receptor tyrosine kinase (19). Active Src phosphorylates the Y576 and Y577 located at the kinase domain of FAK, leading to maximum catalytic activity of FAK, and the formation of a transient FAK–Src signaling complex (17). Cell migration is a key step in metastasis of tumor cells and occurs via two mechanisms: (1) amoeboid, (2) mesenchymal patterns (20). While the amoeboid type of migration has been reported to be independent of integrins and proteases (21), the mesenchymal migration is dependent on integrins, proteases and activation of the FAK signaling pathway (22). In addition, FAK and Src have been associated with migration events such as MMPs expression, secretion and activation which in turn correlates with a highly invasive capacity of tumor cells (23). In particular, MMP-2 and MMP-9 degrade type IV collagen and promote the rupture of basal membranes in colorectal, prostate, lung and breast cancers (24, 25). Importantly, serum samples from breast cancer patients have shown that high levels of MMP-2 and MMP-9 are directly associated with metastasis, and further provide evidence of the participation of these MMPs in breast cancer progression. Considering these evidence, we hypothesized that leptin promotes FAK and Src activation, as well as metastasis-associated events such as cell migration, metalloproteases secretion and invasion.

In this study, we evaluated the role of leptin in the activation of FAK and Src kinases, and their roles in cell migration, metalloproteases secretion, and invasion in a cultured cell model of breast cancer. We utilized the breast cancer cell lines MCF7 and MDA-MB-231 and found that leptin activates FAK and Src. Using a combination of inhibitors for these kinases we found a decrease in metastasis-associated events such as cell migration, metalloproteases secretion, and invasion in breast cancer cells. The data represented here contributes to the molecular characterization of the signaling events associated with leptin contributions to cell migration and invasion in breast cancer cell lines.

## Materials and methods

### Materials

Recombinant human leptin, FAK (PF-573228) and Src (PP2) inhibitors were obtained from Sigma-Aldrich. The Src (SU6656) and the STAT3 inhibitor (S31201) were from Merck. Mouse anti-actin, rabbit anti-FAK and anti-Src antibodies were purchased from Santa Cruz Biotechnology. The phospho-specific antibody against FAK, rabbit anti-pY397, was obtained from Invitrogen. The phospho-specific antibody against Src, rabbit anti-pY418, was obtained from MyBiosource (San Diego, CA, USA), species-specific secondary HRP-conjugated antibodies were from Millipore and the secondary antibody anti-rabbit conjugated with Alexa Fluor 488 was from Invitrogen. Phalloidin coupled to TRITC was purchased from Cytoskeleton (Denver, CO, USA).

### Cell culture

The breast cancer cell lines MCF7 and MDA-MB-231 (ATCC) were cultured in DMEM/F12 media (50:50, V:V; Sigma-Aldrich) supplemented with 5% fetal bovine serum (FBS) and 1% antibiotics (penicillin G/Streptomycin, Gibco) in a humidified atmosphere containing 5% CO_2_ at 37°C. For experimental purposes, cell cultures were serum-starved for 24 h before treatment with either FAK or Src inhibitors and/or leptin. For experimental purposes, cell cultures were used between passages 3 and 15.

### Cell stimulation by leptin and kinase inhibitors

MCF7 and MDA-MB-231 cell cultures were seeded in 60 mm plates containing 4 mL DMEM/F12. When the cell cultures reached confluence, they were washed with PBS, and then treated with FAK (5 µM, PF-573228), Src (5 µM, PP2 and SU6656) or STAT3 (15 and 50 µM) inhibitors, and/or leptin for the times indicated in the figure legends. Stimulation of confluent cells was terminated by removing the medium, and solubilizing the cells in 0.5 mL of ice-cold radioimmune precipitation assay (RIPA) buffer, containing 50 mM HEPES pH 7.4, 150 mM NaCl, 1 mM EGTA, 1 mM sodium orthovanadate, 100 mM NaF, 10 mM sodium pyrophosphate, 10% glycerol, 1% Triton X-100, 1% sodium deoxycholate, 1.5 mM MgCl_2_, 0.1% SDS and 1 mM phenylmethylsulfonyl fluoride (PMSF, Sigma-Aldrich).

### Western blot

Whole cell lysates (20 µg) were resolved on 10% SDS-polyacrylamide gels. Proteins were transferred to nitrocellulose membranes (Bio-Rad). The anti-actin, anti-pY397, anti-FAK, anti-pY418 and anti-Src primary antibodies were incubated overnight at 4°C, in agitation at a 1:1000 dilution. Species-specific secondary HRP-conjugated antibodies (1:5000) were incubated for 2 h at room temperature. Membranes were developed using an enhanced chemiluminescence detection system from Bio-Rad.

### Immunofluorescence

MCF7 and MDA-MB-231 cells were seeded on glass coverslips, grown to 70% confluence, and stimulated with or without leptin for 0, 15, 60 and 120 min. Cells were fixed for 5 min with 4% paraformaldehyde in PBS and permeabilized with 0.2% Triton-X100 in PBS at room temperature. For immunofluorescence (IF) assays, the cells were blocked with 3% albumin in PBS for 1 h at room temperature. Then, the cells were incubated 2 h at room temperature with the rabbit anti-pY397 antibody (1:250 dilution), followed by a 2-h incubation at room temperature with an anti-rabbit conjugated to Alexa Fluor 488 secondary antibody (1:800 dilution). For F-actin staining, cells were incubated with TRITC-Phalloidin (1:500 dilution) for 30 min at room temperature. Cells were counterstained with 4′6-diamidino-2-phenylindole (DAPI), mounted with Fluoroshield/DAPI media (Sigma-Aldrich), and imaged with an Olympus BX43 microscope, using the 100× immersion objective.

### Wound healing assays

MCF7 and MDA-MB-231 cells were grown until confluence on 60-mm culture dishes supplemented with DMEM/F12 as described earlier. Cells were starved for 24 h in DMEM/F12 without FBS and treated for 2 h with Cytosine β-D-Arabinofuranoside (AraC) to inhibit cell proliferation during the experiment. After starvation, cells were scratch-wounded using a sterile 200 μL pipette tip, suspended cells were removed by washing with PBS twice, and the cultures were re-fed with DMEM/F12 in the presence or absence of the FAK and Src inhibitors, and/or leptin as indicated in the figures. The progress of cell migration into the wound was monitored at 0 and 48 h using an Olympus BX43 microscope with a 10× objective. The bottom of the plate was marked for reference, and the same field of the monolayers were photographed immediately after performing the wound (time = 0 h) and 48 h after treatments (time = 48 h), five images per plate were analyzed. The distance between the edges of the wound was measured at time 0 and 48 h, and the reported migrated distance corresponds to the difference between these two. The migration area was determined by measuring the total area of the wound using the ImageJ software and the MRI wound-healing tool (26).

### Gelatin zymography

Cells were stimulated with leptin and the kinases inhibitors as described earlier, the conditioned medium was collected and concentrated using a 30 kDa cut-off ultra-centrifugal filter units (Amicon, Merck-Millipore). Protein was determined by Bradford (27), and 20 µg of protein from concentrated supernatant were assayed for proteolytic activity on native gelatin-substrate gels (28). Briefly, samples were mixed with non-reducing buffer containing 2.5% SDS, 1% sucrose and 4 mg/mL phenol red, and separated in 8% acrylamide gels co-polymerized with 1 mg/mL gelatin, as previously described (29). After electrophoresis at 72 V for 2.5 h, the gels were rinsed twice in 2.5% Triton X-100, and then incubated in 50 mM Tris–HCl pH 7.4 and 5 mM CaCl_2_ assay buffer at 37°C for 24 h. Gels were fixed and stained with 0.25% Coomassie Brilliant Blue G-250 in 10% acetic acid and 30% methanol. Proteolytic activity was detected as clear bands against the background stain of undigested substrate in the gel. Quantification was performed using ImageJ software (26).

### Cell invasion assays

Matrigel invasion assays were performed following the Transwell chamber method (30), using 24 well plates containing inserts of 8 mm pore size (Corning Inc, Kennebunk, ME, USA). Briefly, 30 µL of Matrigel (Corning) was added into the inserts and kept at 37°C for 30 min to form a semisolid matrix. Control and cells treated with the FAK or Src inhibitors were plated at 1 × 10^5^ cells per insert in serum-free medium on the top chamber. The lower chamber of the Transwell contained 600 µL DMEM supplemented or not with 100 ng/mL leptin. Cells were incubated for 48 h at 37°C in a 5% CO_2_ atmosphere. Following incubation, cells and Matrigel on the upper surface of the Transwell membrane were gently removed with cotton swabs. Invading cells on the lower surface of the membrane were washed and fixed with methanol for 5 min, and stained with 0.1% crystal violet in PBS. Cell quantification was performed using a hemocytometer and an Olympus BX43 microscope with the 100× objective.

### Statistical analysis

Results are expressed as mean ± s.d. Data were statistically analyzed using one-way ANOVA and the pairwise comparisons were performed using Newman–Keuls and Dunnett’s multiple comparison test. Statistical probability of *P* < 0.05 was considered significant.

## Results

### Leptin induces FAK activation through the kinase Src in breast cancer cells

**Figure 1:**
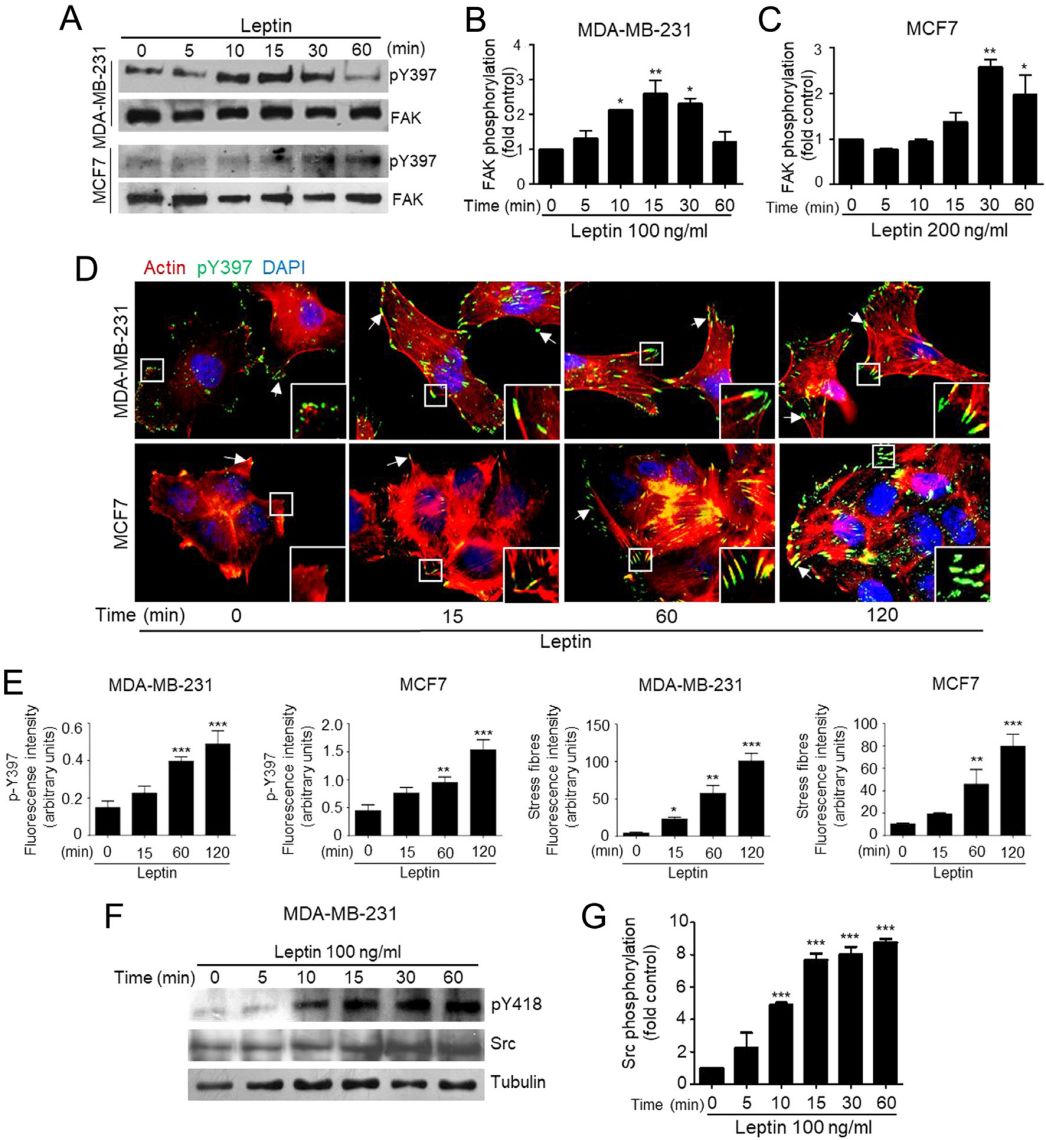
Activation and subcellular localization of FAK in leptin-stimulated breast cancer cell lines. (A) MDA-MB-231 and MCF7 cells were treated with 100 ng/mL and 200 ng/mL of leptin, respectively, for different times and cultures were lysed. Representative Western blots showing phosphorylated Y397 FAK and total FAK were detected using specific antibodies. The plots represent the densitometric and statistical analysis of the bands obtained by Western blot for MDA-MB-231 (B) and MCF7 (C); the values are the mean ± s.d. of at least three independent experiments and are expressed as changes with respect to the control (unstimulated cells). Asterisks denote comparisons made to unstimulated cells. *P < 0.05, **P < 0.01 by one-way ANOVA (Dunnett test). (D) Representative fluorescence microscopy images of MDA-MB-231 and MCF7 treated with or without 100 ng/mL leptin and 200 ng/mL at different time points. Anti-pY397 FAK staining is in green, TRITC-phalloidin is shown in red and DNA is stained with DAPI. Arrows indicate focal adhesions. (E) Quantification and statistical analyses of fluorescent signal of pY397 and stress fiber formation from fluorescence images obtained from MDA-MB-231 and MCF7. Asterisks denote comparisons made to unstimulated cells. *P < 0.05, **P < 0.01, ***P < 0.001 by one-way ANOVA (Dunnett test). (F) Representative Western blot of MDA-MB-231 cells treated with 100 ng/mL leptin at different time points. Phosphorylated Src was detected using an anti-pY418 as well as total Src. (G) Densitometric and statistical analysis of three independent Western blot analyses of phosphorylated Src at pY418. Data represents the mean ± s.d. and are expressed as changes with respect to the un-treated control. Statistical significance was established at ***P < 0.001 by Dunnett test.

FAK activation is triggered by the autophosphorylation of Y397, which is an indicative of its catalytic activity (22, 31). This process has been associated with cancer progression and metastasis-related events (31). First, we performed time-course assays to establish whether leptin induces FAK phosphorylation and activation in MDA-MB-231 and MCF7 breast cancer cell lines. We found that MDA-MB-231 cells treated with 100 ng/mL of leptin present a significant increase in FAK phosphorylation on Y397, after 10 min of treatment and peaked after 15 min compared to the non-treated control cells (Fig. 1A and B). Leptin also activates FAK in MCF7 cells, as Y397 phosphorylation is triggered after 15 min of treatment, reaching a maximum phosphorylation peak at 30 min (Fig. 1A and C). These data indicate that leptin-induced phosphorylation at Y397 occurs shortly after the stimulus, but is not sustained for long periods in both breast cancer cell lines.

FAK activation is associated to the formation of focal adhesions and cell migration, as it contributes to the weakening of cell-cell adhesion and the cyclic assembly-disassembly of focal adhesions (32). Therefore, we analyzed the effect of leptin in the subcellular localization of phosphorylated FAK at Y397, and whether its activation would promote the formation of stress fibers in MDA-MB-231 and MCF7 cells. Cells were incubated with leptin for 15, 30 and 60 min and formation of stress fibers was monitored by confocal microscopy. Optical planes taken in proximity of the cells with the substrate showed that under normal conditions, the MDA-MB-231 cells expressed activated FAK at the periphery of the cells, which increased upon leptin stimulus (Fig. 1D, in green). In these cells, the cytosolic actin filaments were also abundant, and also increased upon the treatment of leptin (Fig. 1D, in red). Interestingly, the effect of leptin in FAK activation, and formation of stress fibers was more evident in MCF7 cells. Fluorescense microscopy analyses of MCF7 cells showed low levels of activated FAK at the periphery of untreated cells (Fig. 1D, in green), which increased throughout the treatment with leptin. Importantly, these cells also had increased formation of stress fibers upon leptin stimulation (Fig. 1D, in red). Quantification of the fluorescent signal for FAK localization at focal adhesions confirmed the increase of this kinase at these regions (Fig. 1E). These results suggest that leptin promotes the phosphorylation of FAK at Y397 and induces the formation of focal adhesions enriched with stress fibers.

**Figure 2:**
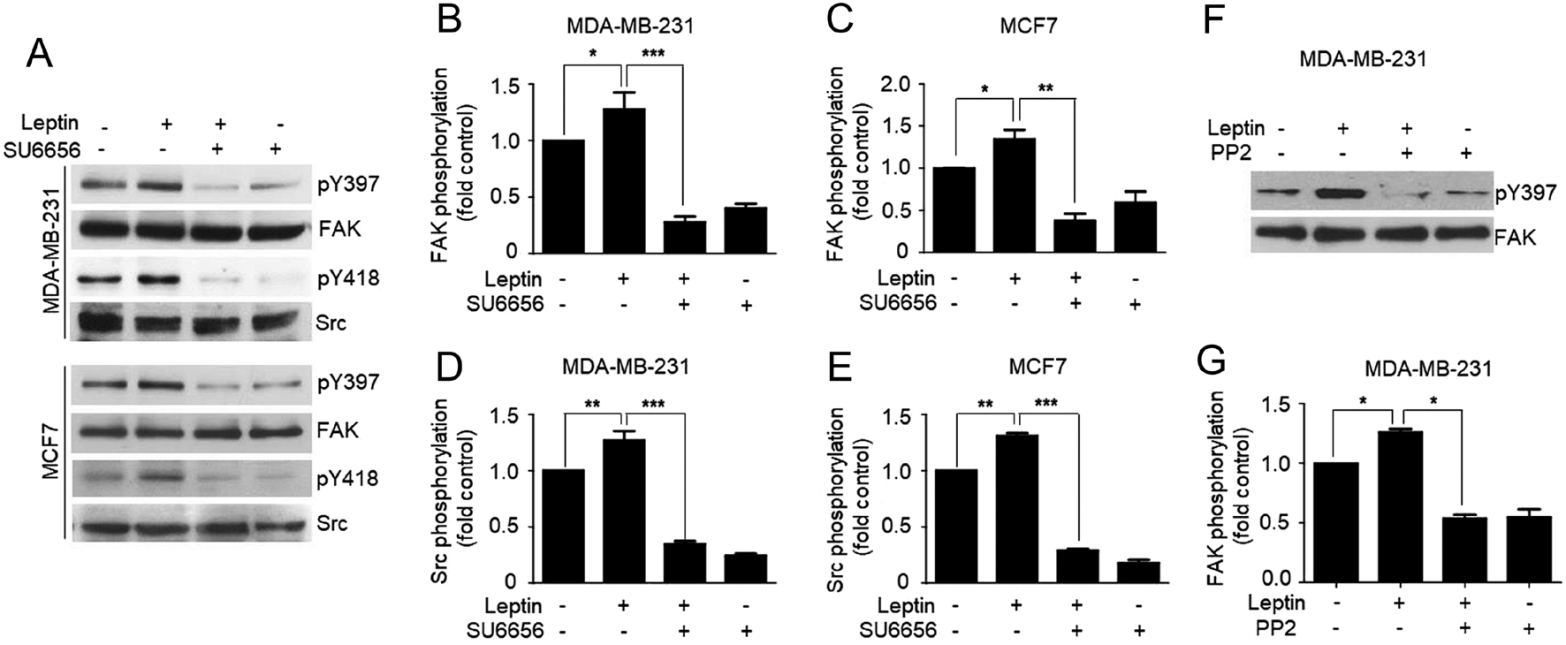
Leptin-induced FAK activation occurs in a Src-dependent manner in MDA-MB-231 and MCF7 breast cancer cells. (A) Representative Western blot of FAK activation at Y397 in the presence or absence of 100 and 200 ng/mL leptin and 5 μM of the Src inhibitor SU6656 in MDA-MB-231 and MCF7 cells. Plots represent the densitometric statistical analyses for each cell type for FAK phosphorylation (B and C), and Src (D and E), respectively. (F) Representative Western blot of FAK activation at Y397 in the presence or absence of 100 ng/mL leptin and 5 μM of the Src inhibitor PP2 in MDA-MB-231 cells. Plots represent the densitometric statistical analyses for each cell type for FAK phosphorylation (G), and Src (H). Antibody against total FAK was used as loading control.

The classic mechanism for FAK activation occurs through autophosphorylation at the Y397 creates a high-affinity binding site for Src (22, 33). The interaction between Src and FAK leads to the phosphorylation and activation of Src at Y419, which further result in the phosphorylation of FAK at Y576 and Y577 (22). Src is a tyrosine kinase involved in cell migration and invasion in different triple-negative cancer cell lines (34). Therefore, we asked whether leptin also played a role in the activation of Src, using the highly invasive triple-negative cell line MDA-MB-231 as an *in vitro* model. Figure 1F shows that leptin induces Src phosphorylation at Y418 in a time-dependent manner, beginning after 10 min of stimulation and is sustained for at least 1 h. Quantification of phosphorylated Src at Y418 further confirmed the activation of this kinase by leptin (Fig. 1G). Then, we asked whether Src was involved on the leptin-induced activation of FAK in both cancer cell lines. To explore this possibility we inhibited Src with the specific inhibitors PP2 (Fig. 2A, B, C, D and E) and SU5566 (Fig. 2F, G, H, I and J). Western blot analysis and quantification showed that Src inhibition in fact decreases FAK activation dependent on leptin in both cancer cell lines. Western blot analyses of phosphorylated Src at Y418 showed that the kinase was efficiently inactivated by the chemical inhibitors (Fig. 2A, D and E). The data are in agreement with previous reports of FAK requirement to promote cell migration in MDA-MB-231 and MDA-MB-468 in breast cancer cell lines (35), and with the effect of FAK autophosphorylation at Y397 in cell migration, invasion, and proliferation of gastric carcinomas (36).

### Leptin promotes cell migration in a FAK/Src-dependent manner and the STAT3 canonical signaling pathway of the leptin receptor in triple-negative breast cancer cell line

**Figure 3:**
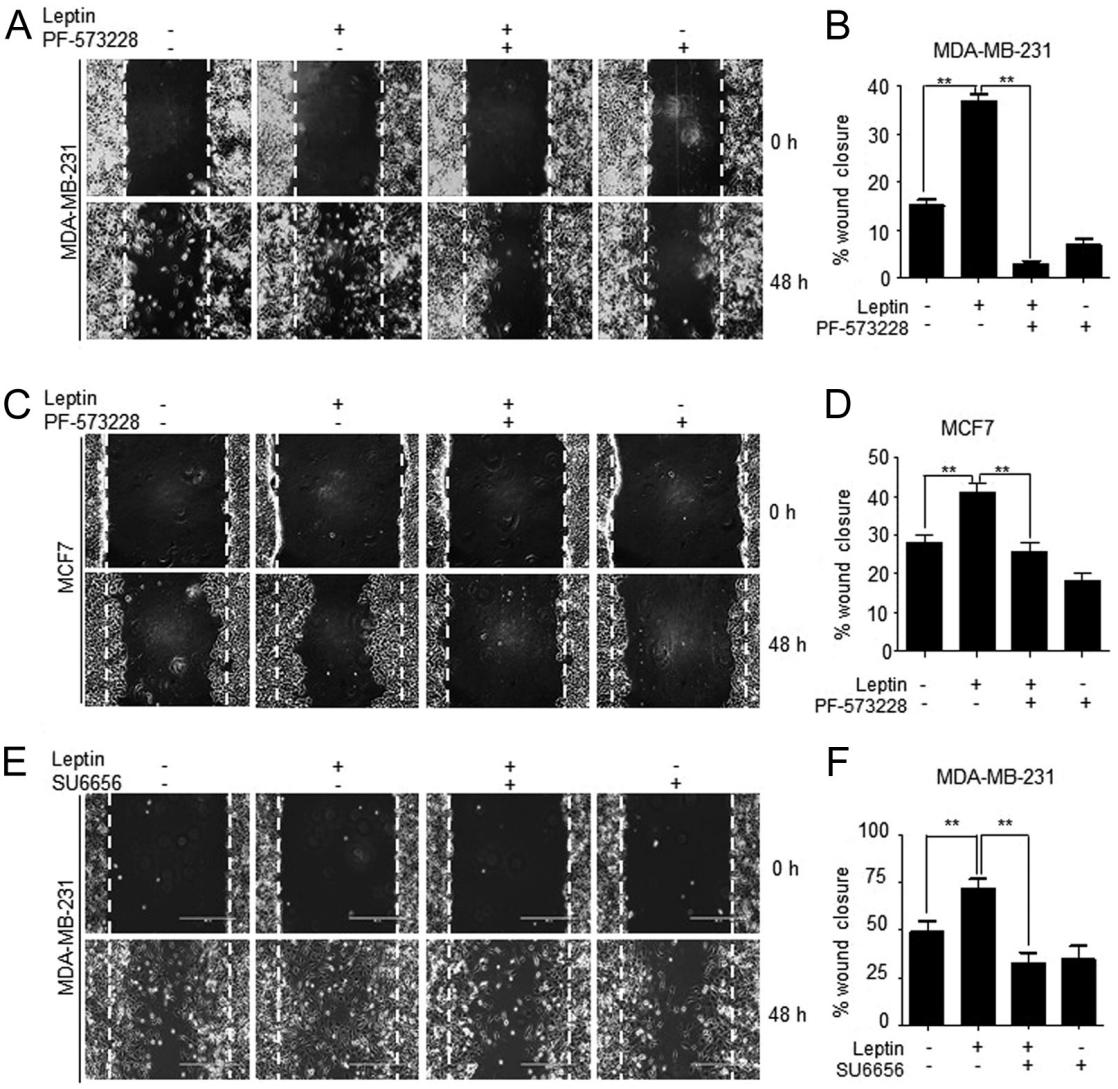
FAK/Src are required for migration of leptin-stimulated MDA-MB-231 and MCF7 breast cancer cells. Confluent monolayers of MDA-MB-231 cells were treated for 2 h with AraC to inhibit proliferation, and then pre-treated with 5 µM of the FAK inhibitor, PF-57322, or with 5 µM of the Src inhibitor, PP2, for 30 min. Wound-healing assays were performed by scratching the monolayer using a sterile pipette tip. Cells were washed and re-fed with DMEM supplemented with or without leptin. The progress of cell migration into the wound was registered at 0 (upper panel) and 48 h (lower panel). Representative light microscopy images and quantification of wounding assays in MDA-MB-231 (A and B) and MCF7 (C and D) breast cancer cell lines and effect of FAK inhibition in the wound closure. (E) Representative light microscopy images and quantification of wounding assays in MDA-MB-231 using SU6656, an Src inhibitor (F) and quantification of the wound closure and the effect of Src inhibition. Data are expressed as percentage of wound closure for each condition and represents the mean of three independent experiments ± s.d. The Newman–Keuls test compares all experimental conditions to determine statistically significant differences; **P < 0.01 by one-way ANOVA.

The increased expression of phosphorylated Y397 FAK at focal adhesion points of MDA-MB-231 and MCF7 cells (Fig. 1) is consistent with the requirement for the cycling of focal adhesions during cell migration (37). These observations prompted us to investigate whether FAK was part of the effect of leptin in the migration of these cancer cell lines. Figure 3 shows representative light microscopy images of MDA-MB-231 and MCF7 cells subjected to wound-healing assays. The data presented at time 0 h are images of the wounds produced at the moment of scratching, and then 48 h after performing the wound depicts the migration of both cell lines. Morphological changes were observed at the front edge of the wound of both cell lines treated with leptin. Leptin induced a mesenchymal phenotype, while the leptin-treated cells cultured with the specific inhibitors of FAK (PF-573228, Fig. 3A and C) and Src (SU6656, Fig. 3E) presented an epithelial phenotype. Quantification of cell migration for both cell lines showed that cells treated with leptin migrated significantly faster than non-treated control cells (Fig. 3B, D and F). However, in the leptin-stimulated MDA-MB-231 cell line (Fig. 2A and B) presented enhanced migration capabilities, compared to MCF7 cells (Fig. 3A and C). Moreover, the MDA-MB-231 cells pre-treated with either FAK or Src inhibitors plus leptin showed a significant decrease in cell migration compared to the leptin-treated cells (Fig. 3A, B, E and F). A similar phenotype was observed in MCF7 cells treated with the FAK inhibitor PF-573228; however, these cells migrated less compared to MDA-MB-231 (Fig. 3C and D). MCF7 cells presented approximately a 30% decrease in the leptin-induced migration properties when treated with the chemical inhibitor of FAK, compared to the leptin-treated cells (Fig. 3A, B, C and D). These results suggest that leptin-induced migration is dependent on the activation of FAK and Src signaling pathways in both cancer cell lines.

**Figure 4:**
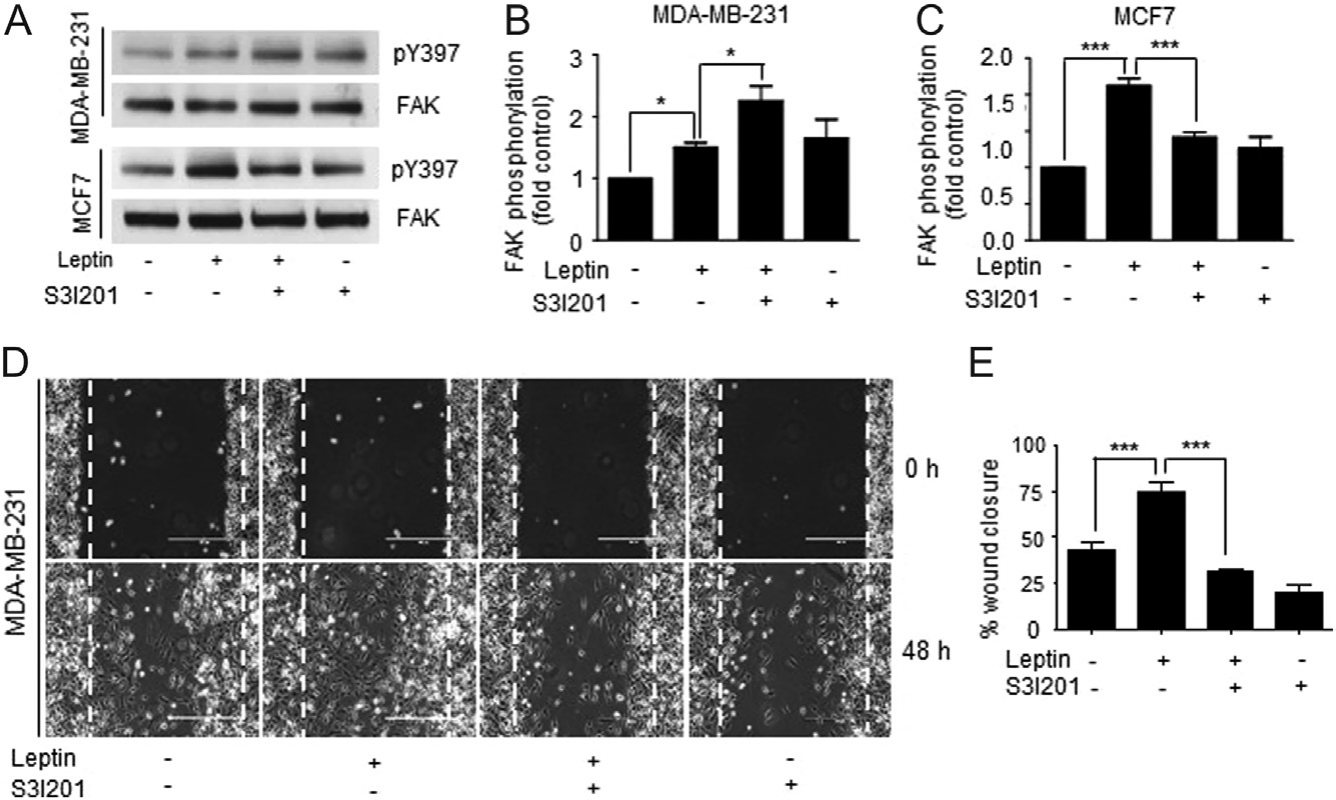
Leptin promotes cell migration and FAK activation independently of the canonical leptin receptor in a model for triple-negative breast cancer cells. (A) Representative Western blot of FAK activation at Y397 in the presence or absence of 100 ng/mL and 200 ng/mL leptin and 50 and 15 μM of the STAT3 inhibitor S3I201 in MDA-MB-231 and MCF7 cells. Plots represent the densitometric statistical analyses for FAK phosphorylation in MDA-MB-231 (B) and MCF7 cells (C). Confluent monolayers of MDA-MB-231 cells were treated for 2 h with AraC to inhibit proliferation, and then pre-treated with 50 µM of the STAT3 inhibitor, S3I201 for 24 h. Wound healing assays were performed by scratching the monolayer using a sterile pipette tip. Cells were washed and re-fed with DMEM supplemented with or without leptin. The progress of cell migration into the wound was registered at 0 (upper panel) and 48 h (lower panel). (D) Representative light microscopy images and (E) quantification of wounding assays in MDA-MB-231 breast cancer cells and effect of STAT inhibition in the wound closure. Data is expressed as percentage of wound closure for each condition and represents the mean of three independent experiments ± s.d. The Newman–Keuls test compares all experimental conditions to determine statistically significant differences; ***P < 0.001 by one-way ANOVA.

Then, we investigated whether the effects of leptin on FAK and Src activation and the enhanced migratory capabilities of cultured cancer cells were mediated by the canonical leptin-receptor signaling pathway. To this end, we focused on the signal transducer and activator of transcription 3 (STAT3), a transcription factor which is activated upon leptin binding to its receptor (7). STAT3 is overexpressed and constitutively activated in triple-negative breast cancer cells and contributes to a wide variety of cellular processes involved in cancer progression (38). Experiments using S31201, the specific inhibitor for STAT3, showed that the leptin-dependent phosphorylation of FAK at Y397 is different between the two cell lines. MDA-MB-231 cells presented higher levels of FAK phosphorylated at the Y437 residue (Fig. 4A and B), while in MCF7 cells this phosphorylation was significantly reduced compared to control cells (Fig. 4A and C). Surprisingly, the higher level of FAK activation upon STAT3 inhibition did not correlated with the enhanced migratory capabilities of these cancer cells observed before (Fig. 3). In this case, the MDA-MB-231 cells treated with leptin and the STAT3 inhibitor, S3I201, presented a significant decrease wound closure compared to leptin-treated cells (Fig. 4D and E). These data suggest that FAK activation is not related to STAT3 signaling pathway, supporting the hypothesis that two independent mechanisms for signal transduction are activated upon leptin stimulation.

### Leptin increased metalloprotease-2 and -9 secretion and activation in breast cancer cells

**Figure 5:**
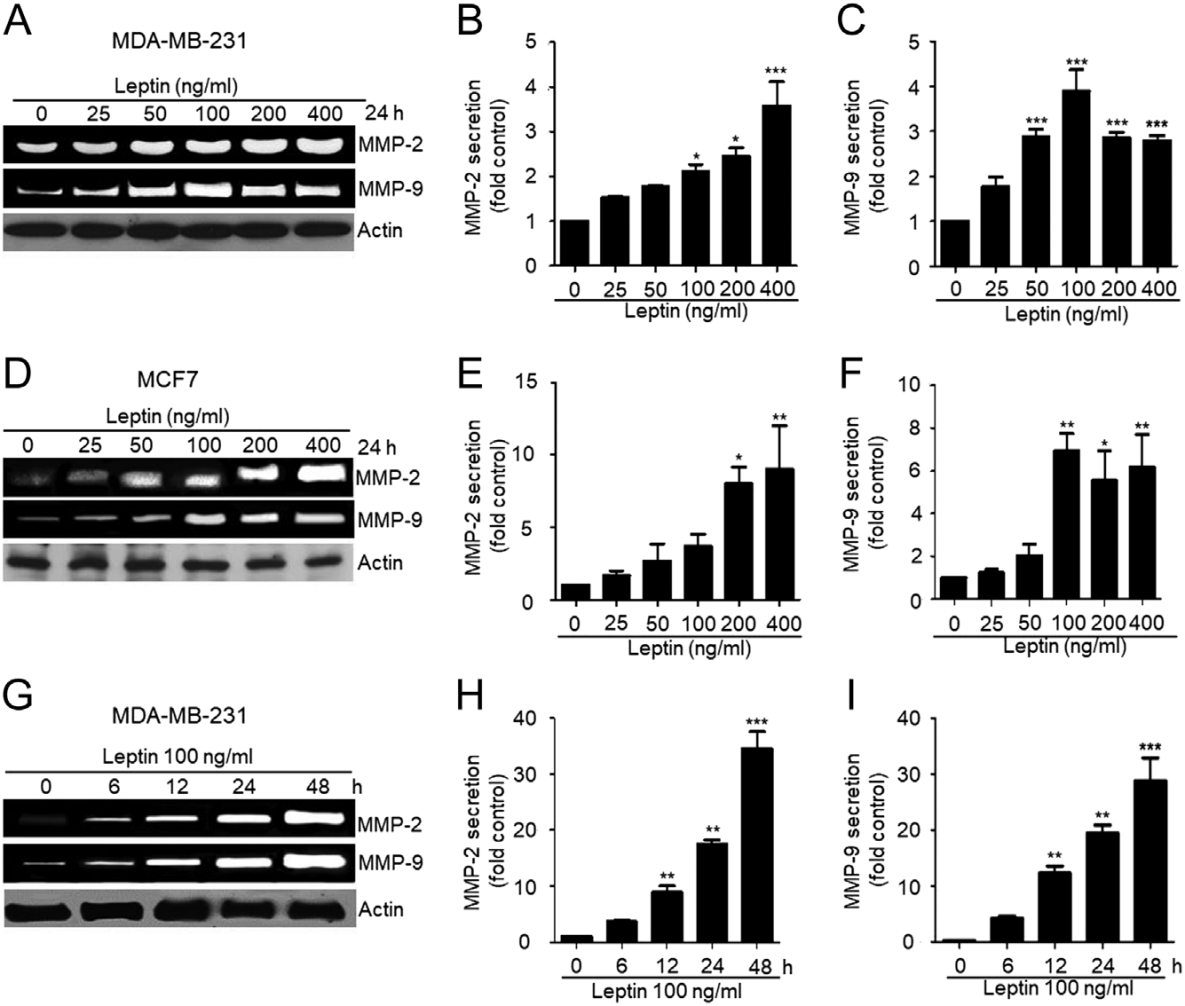
Leptin promotes the secretion of MMP-2 and MMP-9 in the MDA-MB-231 and MCF7 cancer cell lines. (A) Representative zymogram of the gelatinase activity of secreted MMP-2 and MMP-9 obtained from supernatants of MDA-MB-231 cells stimulated with increasing concentrations of leptin for 24 h. Quantification of the gelatinase activity of both secreted metalloproteases MMP-2 (B), MMP-9 (C) under each experimental condition. (D) Representative zymogram of the gelatinase activity of secreted MMP-2 and MMP-9 obtained from supernatants of MCF7 cells stimulated with increasing concentrations of leptin for 24 h. Quantification of the gelatinase activity of both secreted metalloproteases MMP-2 (E), MMP-9 (F) under each experimental condition. (G) Representative zymogram of the gelatinase activity of secreted MMP-2 and MMP-9 obtained from supernatants of MDA-MB-231 cells chronically stimulated with 100 ng/mL of leptin. Quantification of the gelatinase activity of both secreted metalloproteases MMP-2 (H), MMP-9 (I) under each experimental condition. Supernatant samples were collected at the indicated time points. Actin was used as loading control in all cases. Data represent the mean of three independent biological experiments ± s.d., and it is expressed as fold increase over the un-treated control. Statistical significance was established at *P < 0.05, **P < 0.01, ***P < 0.001 by Dunnett test.

During migration, the cells need to modify the composition of the ECM by secreting MMPs. MMPs are secreted in response to growth factors, hormones, and cytokines (39). To be active, the MMPs bind to divalent cations, such as Zn^2+^ (40). MMPs are required for cell migration, wound healing, tissue remodeling, and angiogenesis among other processes, features by which MMPs are highly relevant in diseases such as arthritis and cancer (41). Metalloproteases secretion, in particular MMP-2 and MMP-9, is linked to invasive and metastatic properties of cancers cells (42). Therefore, we asked whether leptin induces the secretion and activation of MMP-2 and MMP-9. To test this hypothesis, MDA-MB-231 (Fig. 5A, B and C) and MCF7 cells (Fig. 5G, H and I) were stimulated with increasing concentrations of leptin for 24 h. The supernatants were collected, concentrated and analyzed by gelatin zymograms to determine the activity of both MMPs. Figure 5 shows that leptin induced a gradual increase in MMP-2 and MMP-9 secretion and activation, which co-related with the amount of leptin supplemented into the culture media. In both cancer cell lines, a significant increase in the activity of MMP-2 and MMP-9 was observed. In the case of MDA-MB-231 cells, the major change in activation of MMP-2 occurred at 100 ng/mL of leptin, while the activity of MMP-9 was significantly higher at 50 ng/mL compared with the non-treated controls (Fig. 5D, E and F). In the case of MCF7 cells, the activity of MMP-2 was significantly higher at 200 ng/mL of leptin, and for MMP-9 was at 100 ng/mL of the hormone (Fig. 5G, H and I). Both MMPs reached a maximum peak of activation at 400 ng/mL in both cell lines.

To determine the time frame for secretion and activation of the metalloproteases induced by leptin, we collected supernatants of MDA-MB-231 cells stimulated with 100 ng/mL of leptin for 6, 12, 24 and 48 h and assayed for gelatinase activity. Figure 5G, H and I show that leptin induces an increase in both, MMP-2 and MMP-9 secretion after 6 h, reaching a maximum activity peak at 48 h. Together, our data suggest that MMP-2 and MMP-9, two relevant proteins for cell migration and metastatic processes, may be induced and activated by the exposure of chronic and increasing doses of leptin. Secretion and activated of these two MMPs may have a direct co-relation with the enhanced mobility capabilities of these cells observed in our wound-healing assays (Fig. 3).

### Leptin promotes invasion and secretion of metalloproteases in a FAK/Src-dependent manner in MDA-MB-231 cells

**Figure 6:**
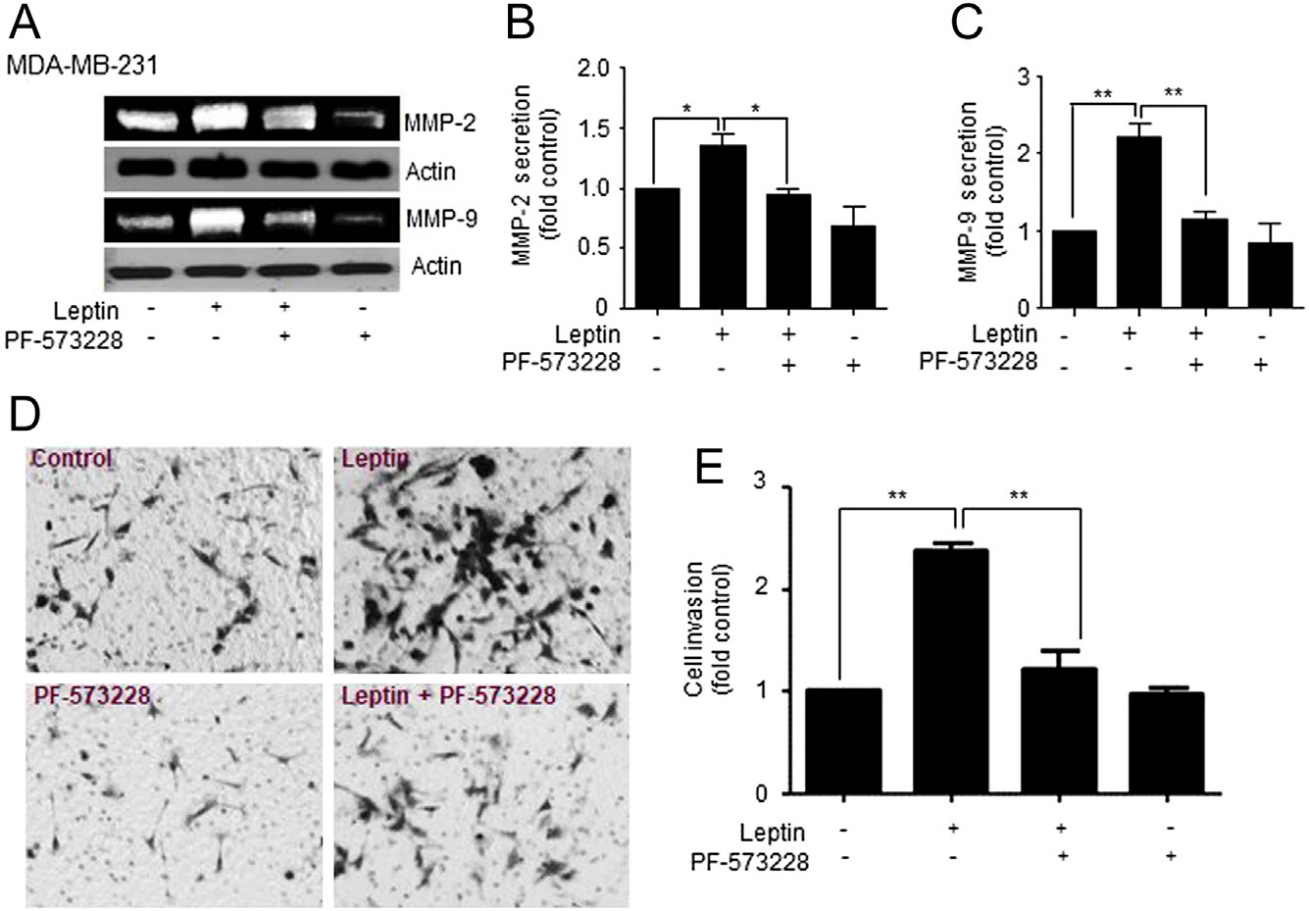
Leptin promotes secretion of metalloproteases and increases an invasive phenotype of MDA-MB-231 cell line in a FAK-dependent manner. MDA-MB-231 cells were pre-treated for 30 min with 5 μM of the FAK-specific inhibitor, PF-573228. Then, the cells were stimulated without or with 100 ng/mL leptin for 24 h. (A) Representative zymogram of the gelatinase activity of MMP-2 and MMP-9 obtained from MDA-MB-231 cells supernatants, actin was used as loading control. Quantification of the activity of secreted MMP-2 (B) and MMP-9 (C) for each experimental condition, and it is expressed as fold increase over the un-treated control. Statistical significance was established at *P < 0.05, **P < 0.01, ***P < 0.001 by Dunnett test. (D) Representative light microscopy images of invasion assays for MDA-MB-231 cells. The cells were pre-treated with 5 μM PF-573228 and seeded at 1x10^5^ cells in Matrigel-coated chambers in the presence or absence of 100 ng/mL leptin for 48 h. (E) Cell invasion quantification represents the number of invading cells for each experimental condition. Data represents the mean ± s.d., and it is expressed as fold increase over the un-treated control. Statistical significance was established at *P < 0.05, **P < 0.01, by one-way ANOVA (Newman–Keuls test).

Metalloproteases secretion is directly related with invasive capacity of tumor cells (43), and might be one of the multiple mechanisms by which FAK positively regulates cell migration (22). If MMP-2 and MMP-9 participate in leptin-dependent migration via FAK activation, their secretion must be controlled by a similar signaling pathway that controls migration of cancer cells. To test this hypothesis, we used the PF-573228 to inhibit FAK signaling pathway and evaluated the effect FAK inactivation on the leptin-induced secretion and activation of MMP-2 and MMP-9. MDA-MB-231 cells were pre-treated with the FAK inhibitor. Then the cells were stimulated with 50 ng/mL of leptin, the supernatants were collected, and concentrated for zymogram analyses. We found that pre-treatment with PF-573228 significantly inhibited MMP-2 (Fig. 6A and B), and MMP-9 (Fig. 6A and C) secretion and activation, when compared to the leptin-treated cells.

**Figure 7:**
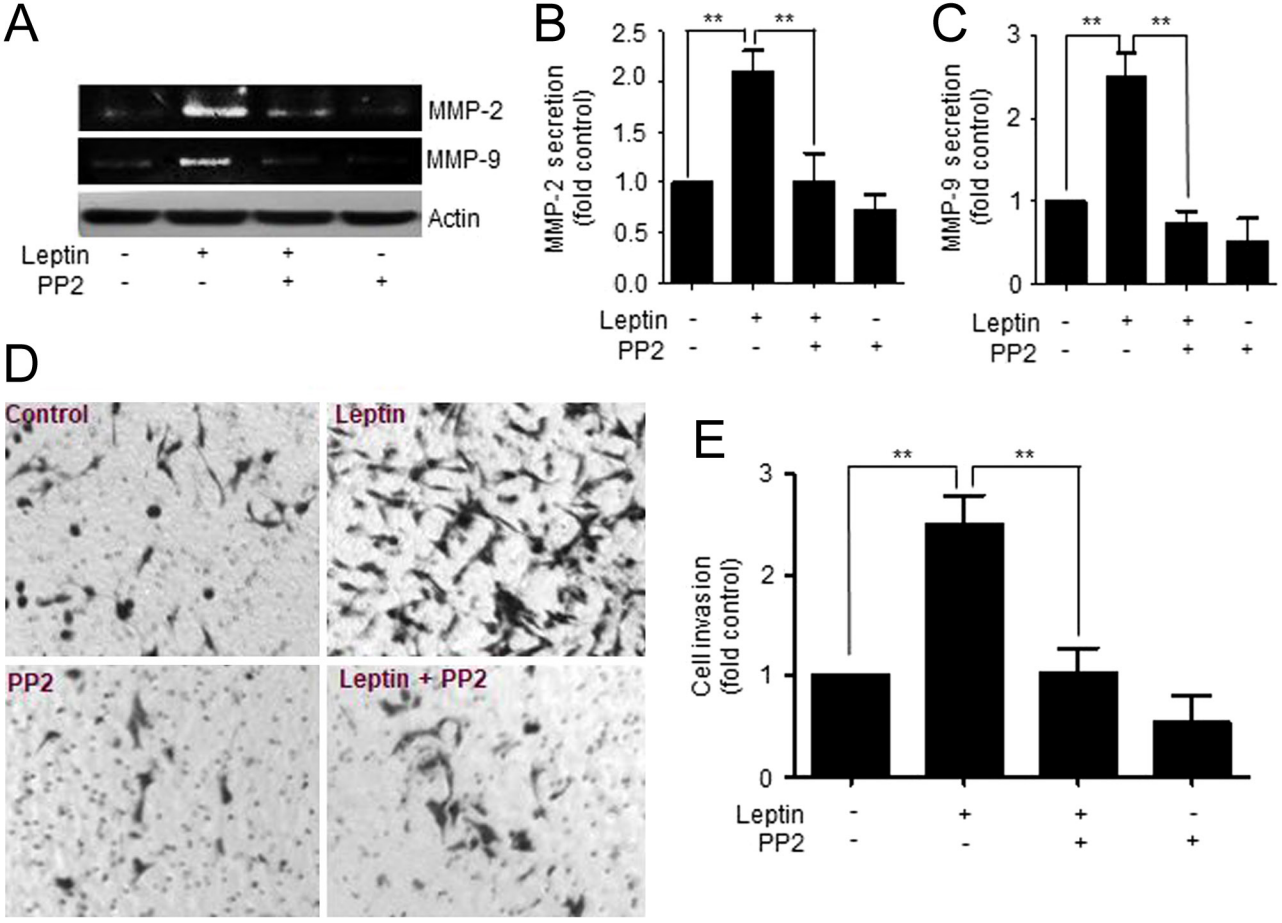
Leptin-induced secretion, activation of MMP-2 and MMP-9 and invasion in MDA-MB-231 cells is Src-dependent. (A) Representative zymogram MMP-2 and MMP-9 obtained from supernatants of MDA-MB-231 cells treated with 5 μM of the Src-specific inhibitor, PP2, and supplemented with or without 100 ng/mL leptin for 24 h. Quantification of the activity of secreted MMP-2 (B) and MMP-9 (C) for each experimental condition, and it is expressed as fold increase over the un-treated control. Statistical significance was established at *P* < 0.05, **P < 0.01, by one-way ANOVA (Newman–Keuls test). (D) Representative light microscopy images of invasion assays for MDA-MB-231 cells. The cells were pre-treated with 5 μM of PP2 and seeded at 1 × 10^5^ cells in Matrigel-coated chambers in the presence or absence of 100 ng/mL leptin for 48 h. (E) Cell invasion quantification represents the number of invading cells for each experimental condition. Data represents the mean ± s.d., and it is expressed as fold increase over the un-treated control. Statistical significance was established at *P < 0.05, **P < 0.01, by one-way ANOVA (Newman–Keuls test).

Further, we tested the role of FAK in the invasiveness capabilities of MDA-MB-231 cells stimulated with leptin. Cell invasion assay allows the evaluation of the intrusive potential of tumor cells through the extracellular matrix, which is directly related to processes related to tumor progression and metastasis (44). Therefore, we sought to investigate whether the cancer cells were able to penetrate a model barrier consisting of components of the basement membrane in response to leptin and FAK inhibition. To this end, the cells were seeded in matrigel-coated Transwell chambers in the presence or absence of 100 ng/ml leptin for 48 h. Figure 7A shows representative light microscopy images of MDA-MB-231 cultured in the presence and absence of leptin, where the hormone promoted an increase in cell invasion, as shown by the presence of darker cells characteristic of cell invasion. Also, we observed that invasiveness is a FAK-dependent process, as the cells treated with 5 μM of PF-573228, showed a decrease in invasiveness properties, compared to cells treated with leptin (Fig. 6D and E). These data is consistent with the contribution of FAK to the highly invasive phenotype of the triple-negative MDA-MB-231 and BT549 cancer cells (35, 45).

**Figure 8:**
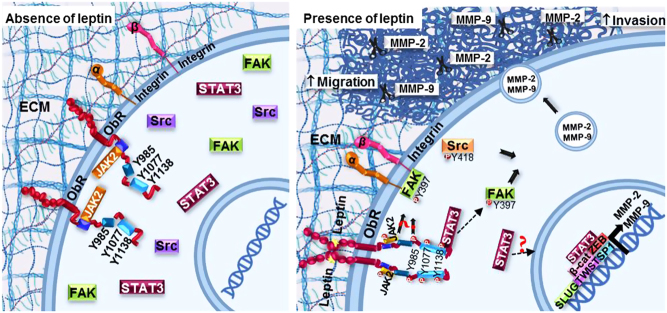
Representative model of the MMP-2 and MMP-9 secretion induced by leptin in a cultured model for breast cancer. Leptin-dependent activation of FAK occurs via Src and an independent mechanism from the canonical ObR pathway. These kinases promote the secretion of MMPs, cell migration and invasion in cultured models for breast cancer cells.

Finally, we evaluated the role of Src in the secretion and activation of metalloproteases in MDA-MB-231 cells. We observed that leptin promotes the secretion of MMP-2 (Fig. 8A and B) and MMP-9 (Fig. 8A and C) through the activation of Src, since culturing the cells with the specific inhibitor of this kinase, PP2, prevented this process. This phenotype was accompanied by a decrease in the leptin-induced invasive capabilities of the MDA-MB-231 cell line treated with PP2 (Fig. 8D and E), supporting the role for Src in cell migration and invasion of cancer cells.

Taken together, our data suggest that leptin activates FAK and Src signaling pathways in a mechanism that is independent to the classic leptin receptor pathway. FAK and Src contribute to the activation and secretion of MMP-2 and MMP-9, which in turn drive the cell migration and invasion phenotypes observed in *in vitro* models of mammary cancer cells. This leptin-dependent phenotype associated to the activation of FAK and Src is consistent with the aggressive phenotype of the tumorigenic and metastatic cancer cells.

## Discussion

Obesity is considered one of the risk factors associated with the development and progression of breast cancer (5, 46). Adipose tissue is characterized by an increased synthesis of different adipokines such as leptin (7). Leptin regulates several physiological functions such as food intake and energy expenditure (8). However, *in vitro* studies demonstrated that leptin also induces the epithelial-mesenchymal transition (EMT), cell migration and MMPs are secreted in the tumor microenvironment in breast epithelial cells (47, 48). Thus, leptin is associated with breast cancer progression (7).

In this study, we used the non-invasive MCF7 breast cancer cell line, and the triple-negative highly invasive MDA-MB-231 to investigate the signaling pathways underlying cell migration and invasion. Our results showed that leptin promotes the phosphorylation and consequent activation of FAK at Y397, in both cell lines; however, the phenotypes observed in the triple negative MDA-MB-231 were greater than those observed in the MCF7 cells. We observed that in both cell lines, leptin activates FAK and Src within 10–15 min of stimulation. Data from the colon cancer cell line SW480 reported by Ratke *et al*. 2010 showed that leptin also induces FAK activation in a time-specific manner with a maximal activation at 15 min, which is consistent with our studies. Although the exact mechanism of leptin-induced FAK activation has not been described, experimental evidence showed that its canonical activation pathway is through the autophosphorylation of Y397 (49), which is in agreement with our observations. This process is a response to the binding of integrins to ECM during the formation of focal adhesions (17). However, this might not be the only pathway for FAK activation. It has been reported that receptors with tyrosine kinase activity may promote FAK activation in NIH 3T3 fibroblastic cell lines (50). Consistently, in our study FAK phosphorylated at Y397 is localized primarily in focal adhesions bound to stress fibers in both breast cancer cell lines, which suggest that this subcellular localization is regulating cell migration. Interestingly, the activation of FAK/Src seems to be independent of the canonical leptin signaling pathway, as inhibition of STAT3, a downstream effector of the ObR, did not prevent the activation of these kinases, but reduced the migratory capabilities of the cells.

We evaluated the effect of FAK and Src on leptin-induced cell migration using the chemical inhibitor PF-573228, and PP2 and SU6656. We found that leptin promotes migration of breast cancer cells in a FAK/Src-dependent manner. Focal adhesions are specialized structures where integrin receptors interact with the ECM, and with the actin cytoskeleton to promote cell migration (51). In addition to regulating the formation of focal adhesions, FAK signaling also affects the remodeling of the actin cytoskeleton by inducing the activation of small GTPases such as Rho, Rac and Cdc42 (52). These are associated to the reorganization of the actin cytoskeleton by generating structures involved in cell migration, such as lamellipodia, filopodia and stress fibers (53).

An important feature of tumor progression in cancer cells is the increased secretion and activation of metalloproteases (54). We focused our studies in MMP-2 and MMP-9 because they are related to invasive and metastatic processes, and a highly invasive capacity of tumor cells (23). Our data showed that in two breast cancer cellular models, leptin induces an increase in MMPs secretion and activation in a time and dose response-dependent manner. These findings are in agreement with evidence collected from serum samples from breast cancer patients, where high levels of MMP-2 and MMP-9 is directly associated with metastasis, and further provide evidence of the signaling mechanisms (55). MMP-2 and MMP-9 expression is connected with different phases of metastasis, such as the pre-metastatic niche formation, formation of new blood vessels and local invasion (56). Furthermore, leptin promoted MMP-2 and MMP-9 and cell invasion of the triple-negative MDA-MB-231 cell line in a FAK/Src-dependent manner. FAK-dependent induction of MMPs is not limited to cancer; in tenocytes the mechano-growth factor promotes MMP-2 secretion and cell invasion in a manner dependent on FAK and ERK kinases (57). MMP-9-dependent degradation of fibronectin also stimulates cell migration, and invasion of MCF-7 cells in a FAK/Src-dependent manner (58). The relevance of FAK/Src in cell migration and invasive processes extends to other cellular systems. For instance, the cell adhesion protein Spondin 1 also promotes cell migration and invasion through FAK and Src activation in human osteosarcoma cell lines (59), and Src is well known to be increase cell permeability, EMT, migration, invasion and metastasis of tumor cells (60). Our data strongly suggest that leptin is associated with the establishment of a more aggressive phenotype of the tumor cells promoting local invasion and eventually metastasis of tumor cells (Fig. 8).

## Conclusion

Taken together, our results demonstrate that leptin promotes cell migration, metalloprotease secretion and invasion in a FAK and Src-dependent manner, which is independent of the classic leptin route. These events partially explain the association between obesity and the development and progression of breast cancer and suggest that kinases FAK and Src are central molecules, regulating events that favor the metastasis of tumor cells stimulated with leptin, promoting changes to a more aggressive phenotype in breast cancer cells.

## Declaration of interest

The authors declare that there is no conflict of interest that could be perceived as prejudicing the impartiality of the research reported.

## Funding

This work was supported by grants from SEP-PROMEP/103.5/14/11118 (UAGro-PTC-053) and SEP-CONACYT CB-2014-01-239870 awarded to N N-T, J C J-C, M O-F, and M D Z-E are supported by a CONACYT Pre-doctoral Training Grant. T P-B is supported by the Faculty Diversity Scholars Award from the University of Massachusetts Medical School.
